# Mechanisms, Detection, and Relevance of Protein Acetylation in Prokaryotes

**DOI:** 10.1128/mBio.02708-18

**Published:** 2019-04-09

**Authors:** D. G. Christensen, J. T. Baumgartner, X. Xie, K. M. Jew, N. Basisty, B. Schilling, M. L. Kuhn, A. J. Wolfe

**Affiliations:** a Department of Microbiology and Immunology, Loyola University Chicago, Health Sciences Division, Stritch School of Medicine, Maywood, Illinois, USA; b Department of Chemistry and Biochemistry, San Francisco State University, San Francisco, California, USA; c Buck Institute for Research on Aging, Novato, California, USA; University of Texas Health Science Center at Houston

**Keywords:** acetylation, acetylome, bacteria, lysine acetyltransferase, mass spectrometry, proteomics

## Abstract

Posttranslational modification of a protein, either alone or in combination with other modifications, can control properties of that protein, such as enzymatic activity, localization, stability, or interactions with other molecules. *N*-ε-Lysine acetylation is one such modification that has gained attention in recent years, with a prevalence and significance that rival those of phosphorylation. This review will discuss the current state of the field in bacteria and some of the work in archaea, focusing on both mechanisms of *N*-ε-lysine acetylation and methods to identify, quantify, and characterize specific acetyllysines. Bacterial *N*-ε-lysine acetylation depends on both enzymatic and nonenzymatic mechanisms of acetylation, and recent work has shed light into the regulation of both mechanisms. Technological advances in mass spectrometry have allowed researchers to gain insight with greater biological context by both (i) analyzing samples either with stable isotope labeling workflows or using label-free protocols and (ii) determining the true extent of acetylation on a protein population through stoichiometry measurements. Identification of acetylated lysines through these methods has led to studies that probe the biological significance of acetylation. General and diverse approaches used to determine the effect of acetylation on a specific lysine will be covered.

## Abstract (precis)

Posttranslational modification of a protein, either alone or in combination with other modifications, can control properties of that protein, such as enzymatic activity, localization, stability, or interactions with other molecules. *N*-ε-Lysine acetylation is one such modification that has gained attention in recent years, with a prevalence and significance that rival those of phosphorylation.

## INTRODUCTION

Protein acetylation occurs across all domains of life, and although it has been well studied in eukaryotes, new insight into the occurrence, mechanism, and relevance of protein acetylation in bacteria and archaea has gained momentum in recent years (1–17). Acetylation is one of many posttranslational modifications (PTMs) that are important in biological systems (18). While prokaryotic acetylation and other acylations were discussed in previous reviews (1, 2, 11, 14, 19–22), due to the rapid pace of advancements in this field, here we will present an updated analysis of prokaryotic protein acetylation. We will highlight various mechanisms of regulation (enzymatic and nonenzymatic), propose a classification system for lysine acetyltransferases (KATs), discuss KAT oligomerization, provide examples of functional relevance for lysine acetylation in the context of bacterial metabolism and other pathways, and describe novel biochemical and analytical tools to identify and quantify acetylation in prokaryotes.

Protein acetylation occurs through a nucleophilic acyl substitution reaction between a nucleophile and an activated acetyl group (CH_3_CO-X), which is typically in the form of acetyl coenzyme A (AcCoA) or acetyl phosphate (AcP) (12). This catalytic reaction can occur either chemically (nonenzymatically) between an acetyl donor and a protein or enzymatically between a protein acetyltransferase, an acetyl donor, and a specific amino acid on a protein substrate. Protein acetylation typically occurs on reactive amino acids containing primary amino groups, hydroxyl groups, or sulfhydryl groups (23–31). Although acetylation of side chains of cysteines, serines, and threonines has been reported, we will focus this review on N-acetylation of primary amino groups (e.g., of lysine).

N*-*acetylation of primary amino groups can be observed either at the alpha amino group (N-α) of N-terminal amino acids or at the epsilon amino group (N-ε) of lysines within a protein. N-α acetylation is very prevalent in eukaryotes, where it is often cotranslational, while it is considered rare and posttranslational in bacteria (7, 14, 32). The prevalence of N-α acetylation varies across archaeal species; however, as in bacteria, it appears to be primarily posttranslational (reviewed in reference 14). The presence of this modification *in vivo* was first discovered by Narita almost 60 years ago (33, 34). Subsequent studies have identified that Saccharomyces cerevisiae may have around 50% of its soluble proteins N-α acetylated (35), while this number is closer to 80% in mammalian cells (36, 37). This is in contrast to bacteria where, for example, 31 proteins have been identified as N-α acetylated in Escherichia coli (38), which accounts for only about 1% of the estimated expressed proteome (39). Since only a few bacterial and archaeal species have been analyzed for N-α acetylation, one should consider that the current collection of proteomic data may be insufficient to indicate whether the reduced prevalence of this modification is the exception or the rule in prokaryotes.

This review will focus on prokaryotic *N*-ε-lysine acetylation, a modification that targets lysine side chains within proteins. First discovered endogenously on histones (40–42), this modification increases the size of the side chain, neutralizes the positive charge of the amino group, and changes protein properties to higher hydrophobicity. Lysine acetylation can alter DNA binding, enzymatic activity, protein-protein interactions, protein stability, or protein localization (43–45). The role of acetylation has been explored extensively in the context of eukaryotic histones where acetylation of disordered tail regions activates gene expression by relieving repression (46, 47). On the other hand, less is known about lysine acetylation in prokaryotes. However, in bacteria it is known that *N*-ε-lysine acetylation can occur either enzymatically or nonenzymatically and that each mechanism appears to be uniquely regulated, as will be described below. *N*-ε-Lysine acetylation is often thought of as reversible through the action of a lysine deacetylase (KDAC); however, in E. coli, most chemical acetylation events do not appear to be reversed by a KDAC (48–50).

### Two types of *N*-ε-lysine acetylation in prokaryotes.

**FIG 1:**
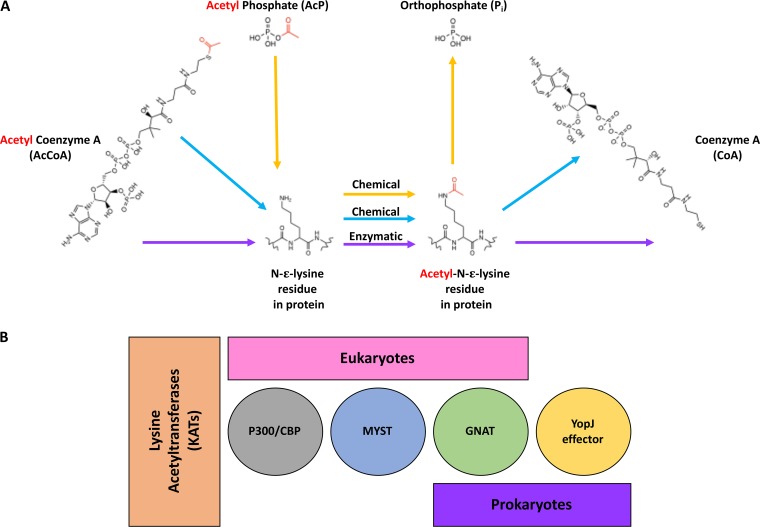
Types of chemical (nonenzymatic) and enzymatic protein acetylation in prokaryotes and comparison of superfamilies of lysine acetyltransferases (KATs) in eukaryotes and prokaryotes. (A) Chemical acetylation of an *N*-ε-lysine of a protein can occur with acetyl phosphate (AcP; yellow arrows) or acetyl coenzyme A (AcCoA; blue arrows), whereas enzymatic acetylation of an *N*-ε-lysine of a protein utilizes AcCoA and a KAT (purple arrows). The acetyl functional group is shown in red. (B) A variety of KATs are found in eukaryotes and prokaryotes, including members of the p300/CBP, MYST, GNAT, and YopJ effector superfamilies. To date, p300/CBP and MYST are found only in eukaryotes, whereas YopJ effectors are found only in prokaryotes. GNATs are found in both eukaryotes and prokaryotes.

**(i) Enzymatic acetylation.** A lysine acetyltransferase or KAT (sometimes called acetylase or transacetylase) catalyzes the targeted transfer of an acetyl group from AcCoA to an epsilon amino group of lysine (Fig. 1A). In eukaryotes, there are several superfamilies of KATs, including p300/CBP (CREB-binding protein), GNAT (Gcn5-related *N*-acetyltransferase), and MYST (named after the founding members MOZ, Ybf2, Sas2, and Tip60), which all acetylate histone and nonhistone protein substrates (51–57) (Fig. 1B). In prokaryotes, two superfamilies of KATs have been identified: GNATs (12, 58, 59) and YopJ effector proteins (60, 61). All KATs use a general acid/base catalytic mechanism to perform their reactions, but the manner in which it occurs differs between families. For example, p300/CBP, MYST, and GNAT proteins typically use an active site glutamate as a general base to deprotonate the amino group of the protein substrate and use an active site tyrosine as a general acid to reprotonate the product of the reaction, coenzyme A (CoA) (62–65). However, some exceptions to this general mechanism have been identified for GNATs. For instance, a water molecule can replace glutamate as a general base in the reaction through a proton wire (62, 66). On the other hand, YopJ effectors utilize histidine to deprotonate a nearby cysteine, which then attacks AcCoA to form an acyl-enzyme intermediate and then transfers the acetyl group to the protein substrate (60). The predominant kinetic mechanism for p300/CBP and GNATs is a sequential/direct transfer mechanism that proceeds through a ternary complex (63, 64, 67). Previously, the MYST family protein Esa1 had been characterized as exhibiting a ping-pong/double-displacement mechanism using an enzyme intermediate (25); however, it was subsequently shown to unambiguously utilize a direct transfer mechanism (65). YopJ effector proteins use a ping-pong/double-displacement mechanism (60).

**FIG 2:**
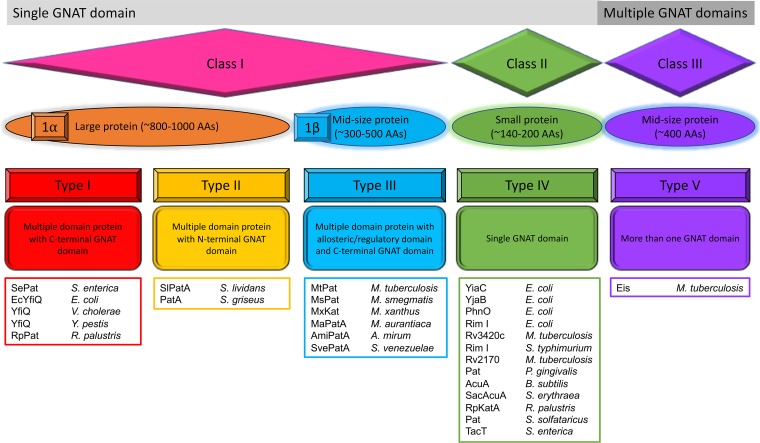
Classification of GNAT KATs in prokaryotes. Prokaryotic KATs can be currently divided into three classes (I, II, and III) based on their size. These classes are further subdivided into types (I to V) based on the number of GNAT domains present in a particular sequence, the arrangement of domains, and type of domain (i.e., allosteric/regulatory or not). Characterized representatives are listed in colored boxes below each type.

Since no standardized naming scheme exists for prokaryotic KATs of the GNAT family, we have combined the schemes of Hentchel and Escalante-Semerena (12) and Lu et al. (68) into one classification system (Fig. 2). Previously, Hentchel and Escalante-Semerena (12) proposed four different types of prokaryotic KATs, whereas Lu et al. (68) proposed two major classes. New evidence exists for KATs with additional domain organization and structures; therefore, we also included an additional class and type of KAT into our proposed system (Fig. 2). In this system, there are three main classes (class I, II, or III) of KATs based on sequence length and number of GNAT domains present and five different types (types I to V) of KATs based on domain identities and arrangements. Class I KATs consist of large multidomain enzymes with a single GNAT domain, and class II KATs are smaller enzymes with only a single GNAT domain, whereas class III KATs have multiple GNAT domains. Class I KATs are further subdivided into class Iα (NDP-forming acyl-CoA synthetase domain and a GNAT catalytic domain) and class Iβ (effector/regulatory domain and a GNAT catalytic domain). Each class of KATs is further categorizated into types. Type I KATs have a C-terminal GNAT domain, whereas type II KATs have an N-terminal GNAT domain; both types belong within class Iα. Type III KATs have an N-terminal regulatory domain and a C-terminal GNAT domain and belong to class Iβ. Type IV and V KATs belong to classes II and III, respectively.

Type I and type II GNATs (class Iα), represented by YfiQ and its homologs (also known as Pat, PatZ, and Pka), are currently the best-studied class of bacterial KATs. YfiQ is a conserved acetyltransferase (12) with homologs found across many bacterial species, including E. coli (69), Vibrio cholerae (5), Salmonella enterica (70), Yersinia pestis (10), Rhodopseudomonas palustris (71), Streptomyces lividans (72), and Streptomyces griseus (73). The best-understood role for YfiQ is acetylation of acetyl-CoA synthetase (Acs); acetylation inactivates Acs, preventing acetate consumption (70). However, the phenotypic effects of YfiQ have been extended to protection against acid stress (74), high temperature (75), and reactive oxygen species (75), albeit by unknown mechanisms. Additional substrates for E. coli YfiQ (EcYfiQ) have also been identified, expanding the number of known protein substrates and potential regulatory roles for this enzyme (76). The oligomeric state of S. enterica Pat (SePat) has been found to be monomeric in the absence of AcCoA, but SePat will tetramerize in its presence. Moreover, the non-GNAT domain of SePat contributes to the oligomerization and activity of the enzyme, and oligomerization is likely the source of positive cooperativity observed in the enzyme (77). EcYfiQ has also been observed to exhibit cooperativity and form a tetramer even in the absence of AcCoA. However, the presence of AcCoA and autoacetylation of three EcYfiQ lysines are required for the formation of an octomer (66).

Type III (class Iβ) GNATs primarily consist of KATs with allosteric regulatory domains, with a wide range of possible effectors (e.g., arginine, cysteine, cAMP, and NADP^+^). Three different types of regulatory domains that are fused to the classical GNAT catalytic domain have been identified for prokaryotic KATs: an amino acid binding domain (ACT), a cAMP binding domain, and an NADP^+^ binding domain (68, 78–80). For instance, ACT domain KATs (AAPatAs) have been discovered in actinobacteria, directly linking acetylation activity with central metabolic pathways. An AAPatA from Micromonospora aurantiaca (MaPatA) was found to exhibit mixed activation kinetics in the presence of cysteine or arginine against Acs (81). Further studies by the same group found a set of AAPatAs unique to actinobacteria that could be broadly divided into Cys-binding AAPatA and Asn-binding AAPatA; example studies performed with Actinosynnema mirum (AmiPatA) Cys-binding and Streptomyces venezuelae (SvePatA) Asn binding found that the presence of the allosteric effector increases the lysine acetylation activity toward AmiAcs and SveAcs, respectively (68). Pats from Mycobacterium smegmatis (MsPat; MSMEG_5458) and Mycobacterium tuberculosis (MtPat; Rv0998) acetylate Acs and are tightly controlled by cAMP (78, 79, 82). Moreover, MxKat from Myxococcus xanthus also acetylates Acs, but it appears to be allosterically inhibited by the binding of NADP^+^ to a noncatalytic NADP^+^ binding domain, which causes an increase in Acs activity. The allosteric regulation of MxKat is linked to the intracellular ratio of NADP^+^ to NADPH (80), reminiscent of the linkage between sirtuin-like deacetylases and the intracellular NAD^+^/NADH ratio (83).

Type IV (class II) GNATs consist of KATs that feature a single catalytic GNAT domain and are typically about 150 to 200 amino acids long, whereas type V (class III) GNATs are midsize proteins (∼400 amino acids) comprised of multiple GNAT domains. A large number of type IV KATs have been identified, but the depth of their characterization in terms of their roles in protein acetylation is less than that of class I GNATs. The same is true for type V KATs, as there is currently only one characterized example in the literature, enhanced intracellular survival (Eis) protein from M. tuberculosis (84–86). Recently, four new type IV GNATs from E. coli were identified (YiaC, YjaB, RimI, and PhnO), and they appear to acetylate a wide range of protein substrates (76). Previously, the M. tuberculosis Rv2170 KAT protein was found to acetylate key lysines on isocitrate dehydrogenase (ICDH), which results in a reduction of ICDH activity (87). Other identified type IV KATs include Porphyromonas gingivalis Pat (88), Bacillus subtilis AcuA (89), Saccharopolyspora erythraea SacAcuA (90), Rhodopseudomonas palustris RpKatA (71), Sulfolobus solfataricus Pat (91, 92), and S. enterica TacT (93).

Interestingly, many type IV (class II) and type V (class III) KATs appear to often be capable of additional activities beyond lysine acetylation. For example, the newly identified KAT RimI (76) had been characterized previously as an *N*-α-acetyltransferase in *Salmonella enterica* serovar Typhimurium (94), E. coli (95, 96), and M. tuberculosis (Rv3420c) (97), while the newly identified KAT PhnO was shown to be an aminoalkylphosphonate acetyltransferase in S. enterica (98) and E. coli (99). Another intriguing example is TacT (STM3651) from S. enterica, the toxin protein of the TacAT toxin/antitoxin system, which acetylates aminoacyl-tRNAs (100) and Lys44 of its own TacA antitoxin pair (93). TacA acetylation also was found to be regulated by the KDAC CobB (93). Another example is Eis from M. tuberculosis. This type V KAT was initially discovered as an aminoglycoside acetyltransferase (101, 102), but it also has protein acetylation activity, acetylating over 30 lysines on an M. tuberculosis nucleoid-associated protein (MtHU or HupB) (84) and acetylating Lys55 of host macrophage dual-specificity protein phosphatase 16/mitogen-activated protein kinase phosphatase-7 (DUSP16/MKP-7) (86). The general broadness of possible substrates suggests that a reexamination of previously characterized GNATs with newly developed techniques for detecting enzymatic acetylation is warranted.

**(ii) Nonenzymatic or chemical acetylation.** In eukaryotic cells and specifically within mitochondria, AcCoA, a high-energy thioester, can nonenzymatically acetylate proteins (103) (Fig. 1A). For example, histones were shown to be acetylated *in vitro* by AcCoA (104). Indeed, under conditions mimicking those within the mitochondrial matrix, many mitochondrial proteins can be nonenzymatically acetylated by AcCoA *in vitro* (105). Mechanistically, mitochondrial nonenzymatic protein acetylation appears to occur through the acetylation of a nearby cysteine by AcCoA followed by the transfer of the acetyl group to a deprotonated lysine or through off-target binding of a thioester near a lysine (106, 107). Nonenzymatic AcCoA-dependent acetylation likely occurs in bacteria as well. Because AcCoA is essential in most organisms, it cannot be removed from the system, and thus, obtaining *in vivo* proof is difficult. However, due to the presence and availability of AcCoA in cells, these nonenzymatic reactions most likely also modify the bacterial proteome (105, 108). Indeed, as shown for mitochondrial proteins, exposure of several purified bacterial proteins to AcCoA alone leads to their acetylation (108–110).

In bacteria, acetyl phosphate (AcP), another high-energy molecule, can nonenzymatically acetylate proteins (49, 50, 111) (Fig. 1A). It is worth noting that AcP has been detected within the mitochondria and thus also may act as an acetyl donor in eukaryotic cells (112). A lysine susceptible to nonenzymatic AcP-dependent acetylation seems to have two important features that depend on the three-dimensional protein structure and not on a linear motif (49). First, the reactive lysine is deprotonated, which can occur through negatively charged amino acids (i.e., Asp or Glu) or by a water molecule. Second, the protein positions AcP to allow nucleophilic attack by the activated lysine. To achieve this, the phosphoryl group of AcP can be coordinated via positively charged amino acids (Lys or Arg), hydrogen bonds from hydroxyls (Ser, Thr, or Tyr), or side chain amide groups (Gln or Asn). Alternatively, if the local pH around a lysine is basic, the epsilon amino group can act as a nucleophile toward AcP. Although not dependent on a specific linear sequence, there is a propensity for glutamate and/or aspartate near the +1 or −1 position relative to an acetylated lysine, which reduces the pK_a_ of lysine to promote activation (49, 108, 111, 113, 114).

### AcP synthesis and relevance of global AcP-dependent acetylation.

**FIG 3:**
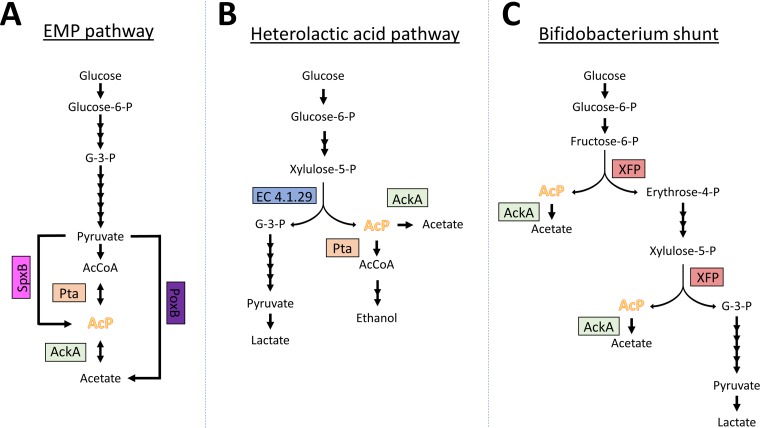
Metabolic pathways that generate AcP. (A) The EMP pathway produces pyruvate, which is converted to AcCoA. AcCoA can be fermented as acetate via the Pta-AckA pathway, through which AcP is made as an intermediate. In E. coli and other bacteria, PoxB (pyruvate oxidase) is expressed in stationary phase and directly converts pyruvate to acetate. Alternatively, in Streptococcus pneumoniae and other bacteria, SpxB (pyruvate oxidase) instead converts pyruvate to AcP. (B) The heterolactic acid pathway found in some lactic acid bacteria uses a phosphoketolase (EC 4.1.29) that cleaves xylulose-5-phosphate into glyceraldehyde-3-phosphate and AcP, which can be converted to ethanol or acetate. (C) The *Bifidobacterium* shunt generates AcP in two separate reactions with the bifunctional phosphoketolase XFP.

In E. coli and multiple other species of bacteria, AcP seems to be the predominant acetyl donor (49, 50, 111). In these species, AcP is generated as an intermediate of the phosphotransacetylase (Pta)-acetate kinase (AckA) pathway (Fig. 3A). This reversible pathway is responsible for acetate fermentation when carbon is in excess but will consume acetate when better carbon sources are unavailable. Pta is a remarkable enzyme, uniquely capable of using inorganic phosphate to convert AcCoA to AcP, releasing CoA. AckA can then convert the AcP to acetate while generating an ATP (115). Interestingly, this is not the only pathway capable of generating AcP. In addition to Pta, E. coli encodes two proteins that contain a phosphotransacetylase PTA-PTB domain, and while EutD and Pta can generate AcP, MaeB cannot (116). EutD is a Pta paralog that converts AcCoA to AcP during ethanolamine catabolism (117). However, induction of the *eut* operon depends on ethanolamine and adenosyl-B_12_ (118) and thus is not transcribed under all conditions. Another enzyme, PurT, has secondary acetate kinase activity. PurT is one of two enzymes that can catalyze the third step of *de novo* purine biosynthesis, but little about its ability to generate AcP has been explored (119). It is also worth noting that other species generate AcP by other mechanisms. For example, *Lactobacillus* and *Streptococcus* species use pyruvate oxidase (SpxB) to synthesize AcP, which is then converted to AcCoA via Pta or to acetate via AckA (Fig. 3A). Note that the AcP-forming pyruvate oxidase SpxB is distinct from the acetate-forming pyruvate oxidase (PoxB) of E. coli that directly produces acetate from pyruvate (115).

Generation of AcP as described above is peripheral to glycolysis and thus nonessential, although deletion of SpxB, PoxB, or the Pta-AckA pathway can have deleterious effects on growth and affects other aspects of physiology (120–126). However, in some Gram-positive species, synthesis of AcP is essential and central to their metabolism. For example, in the unique glycolytic pathways employed by certain *Bifidobacterium* and *Lactobacillus* species, the enzyme phosphoketolase can cleave a pentose sugar into glyceraldehyde-3-phosphate and AcP (Fig. 3B). In another unique glycolytic pathway employed by other *Bifidobacterium* species, the enzyme XFP (xylulose-5-phosphate/fructose-6-phosphate phosphoketolase) synthesizes AcP from two distinct substrates: fructose-6-phosphate and xyulose-5-phosphate (127) (Fig. 3C). It would be interesting to compare the extent of acetylation in these bacteria and the contribution of AcP to bacteria like E. coli and B. subtilis where AcP-induced acetylation is peripheral to glycolysis.

In E. coli, the Pta-AckA pathway accounts for most of the observed global acetylation. This was shown genetically by observing that an Δ*ackA* mutant that accumulates AcP achieves stronger acetylation, while an Δ*ackA pta* mutant that cannot generate AcP achieves very weak acetylation (49, 50). Furthermore, a Δ*pta* mutant normally phenocopies the weak acetylation of an Δ*ackA pta* mutant, but by supplementing this strain with acetate, increased acetylation can occur through AckA-dependent AcP generation (49). Thus, any perturbations of flux through the Pta-AckA pathway will influence the generation of AcP, which can yield AcP levels in the high-micromolar to low-millimolar range (50, 128, 129).

We and others have found that accumulation of global acetylation depends on two major factors: rapid carbon flux and a carbon-nutrient imbalance that restricts growth (50, 130). The Pta-AckA pathway acts as a safety valve for E. coli during a process known as overflow metabolism (also called aerobic fermentation or the bacterial Crabtree effect) (131). When the flux of carbon into the AcCoA node exceeds the capacity of the TCA cycle, fatty acid biosynthesis, and other central metabolic pathways, the cell needs to regenerate limiting CoA pools to continue glycolytic carbon consumption. Thus, AcCoA is hydrolyzed and converted into acetate, which produces AcP and an ATP molecule along the way (115). It is typically underappreciated that when cells are exposed to high carbon, they will globally accumulate acetylation on their proteins, which may have unexpected phenotypic consequences. While proteins become acetylated throughout growth, the generation of nascent proteins keeps the relative amount of acetylation in the population low. However, when a culture enters stationary phase due to reduced availability of a noncarbon nutrient (e.g., nitrogen [50] or magnesium [132]), acetylation can accumulate due to continued metabolism of carbon coupled with reduced nascent protein synthesis until the carbon source is exhausted. As expected, wild-type (WT) cells growing on glucose achieve strong AcP-dependent acetylation, but this acetylation can be reduced or abolished by preventing rapid flux of glucose into the cell, by forcing glucose through alternative transporters, or by preventing rapid flux through glycolysis. This has been achieved by deleting *ptsG*, the gene that encodes the major glucose transporter, or by deleting *pgi*, which encodes the first enzyme in the Embden-Meyerhof-Parnas (EMP) pathway, thereby forcing the mutant cells to use the slower pentose phosphate pathway (130). Acetylation also can be reduced by alleviating the carbon-nutrient imbalance (132).

### Deacetylation.

While acetylation occurs either enzymatically or nonenzymatically, removing an acetyl group requires a lysine deacetylase (KDAC). Two major families of KDACs have been discovered: the zinc-dependent Rpd3/Hda1 family (133) and the NAD^+^-dependent sirtuin family (134). Both of these classes can be found across bacteria and archaea. In E. coli and S. enterica, the only known KDAC is the sirtuin CobB (48). While YcgC of E. coli was proposed to be a KDAC (135), this was found to be incorrect (136). Further suggesting that CobB is the sole known deacetylase in E. coli, a Δ*cobB* mutant showed almost no deacetylase activity against an acetylated peptide library (48). Depending on the conditions, the number of acetylated targets regulated by CobB *in vivo* is between 5% and 14% (49, 50, 114), which is dwarfed by the number of targets regulated by YfiQ (76) or AcP (130).

Importantly, CobB can deacetylate acetyllysines generated via AcP and YfiQ (69, 137–140). Since CobB deacetylates only a fraction of YfiQ-dependent acetyllysines and can also deacetylate selected AcP-dependent acetyllysines, it is probably incorrect to call CobB and YfiQ/Pat a system (12, 141, 142); instead, these enzymes appear to function independently. Indeed, it is difficult to imagine how CobB could distinguish a YfiQ-dependent acetyllysine from an AcP-dependent acetyllysine unless YfiQ and CobB form a complex for which there is no evidence.

The determinants for acetyllysine susceptibility to CobB are not obvious. Its substrates tend to be surface exposed on α-helices and loops. Perhaps the selectivity of CobB for certain substrates allows for greater promiscuity for removing various modifications. Indeed, CobB may be a deacylase capable of removing, in addition to acetyl groups, succinyl (143), propionyl (144, 145), lipoyl (146), and homocysteine (147) functional groups.

**FIG 4:**
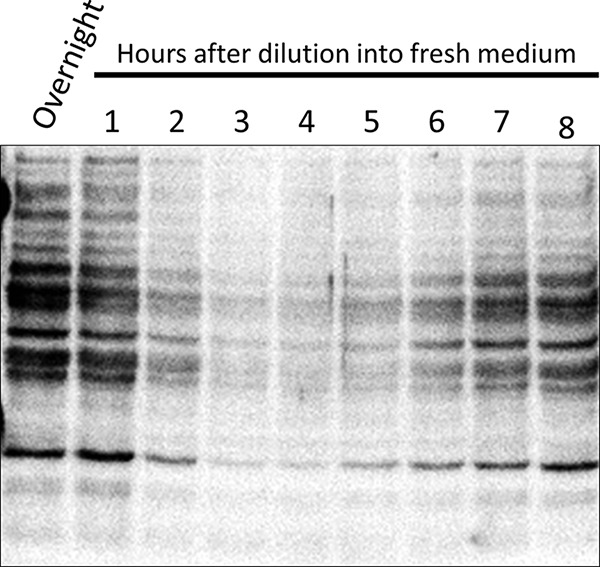
Global acetylation is diminished in exponentially growing cells. Cells were grown overnight in M9 minimal medium supplemented with 0.4% glucose (lane 1) and subsequently diluted into fresh M9 supplemented with 0.4% glucose. Samples were harvested hourly, normalized for protein concentration, and analyzed by antiacetyllysine Western blot assay as described previously (49, 130).

Not all acetyllysines are sensitive to CobB or the corresponding deacetylase in other organisms. There could be another class of deacetylase that is not yet characterized, but, even if there were, acetylated lysines have been found buried within folded proteins, such that it would be unlikely for that acetylation to be reversed enzymatically. For example, the first ribonucleotide of an RNA message is oriented by a lysine within RNA polymerase. This lysine is sensitive to AcP-dependent acetylation, a modification that would be both inactivating and irreversible (49). We propose that deacetylation of such buried acetyllysines does not readily occur, but on a population level “deacetylation” occurs through protein turnover and/or dilution, i.e., synthesis of new proteins replaces acetylated isoforms that can and cannot be reversed. Exponential-growth-phase cells must synthesize enough proteins for two daughter cells. This would dilute acetylated isoforms 2-fold every division (148), resulting in an exponential dilution of any proteins that either were previously acetylated or become acetylated during growth. Indeed, these nonacetylated proteins seem to become the most prevalent isoforms when comparing acetylations of exponential-growth-phase cells diluted from highly acetylated stationary-phase cells (Fig. 4). Alternatively, deacetylation may be possible if previously buried acetyllysines become exposed when certain proteins undergo conformational flucations, sometimes called “breathing” (149). However, the molecular crowding of the cytoplasm may prevent such movements and thus restrict this possibility (150).

### Mass spectrometry to study protein acetylation.

**(i) Identification and qualitative assessment of acetylation.** To study acetylation in an organism, it is important to generate sensitive and appropriate analytical methods and protocols to detect and quantify lysine acetylation. A simple and effective method is to perform an antiacetyllysine (K^ac^) Western blot analysis of proteins harvested from the organism of interest using commercially available anti-K^ac^ antibodies. While neither exquisitely sensitive nor site specific, Western blots can show qualitative differences between strains, growth phases, and environmental conditions. Using E. coli as an example, Western blots were used to reveal that AcP is the predominant acetyl donor by comparing mutants that accumulate AcP (Δ*ackA*) to those that cannot generate AcP (Δ*ackA pta*) (49). Data from a Western blot can guide experimental design for more time- and cost-intensive approaches like mass spectrometry. The Western blot depends on the eponymous anti-K^ac^ antibody. However, it appears not all of these antibodies are equal. In a recent study that compared an anti-K^ac^ antibody from Abcam to one from Cell Signaling Technology (CST), the Abcam antibody was more discerning than the CST antibody (136). While the CST antibody showed robust staining of RutR under all conditions, the Abcam antibody detected only site-specifically acetylated RutR and could detect deacetylation after CobB treatment. Possible explanations for these discrepancies include differential sensitivities, cross-reactivity to RutR, or the possibility of a bad batch of antibody. Thus, it is important to verify detection of K^ac^ via whichever antibody is chosen by using a commercially available acetylated protein, such as BSA, and confirming the results by mass spectrometry whenever possible.

**(ii) Mass spectrometry enables identification and quantification of specific lysines.** There has been a rapid bloom in techniques to study PTMs by mass spectrometry (151). Depending on the biological interest, mass spectrometry can be employed to study PTMs either in a targeted way or in a global manner. With high-resolution mass spectrometry-based proteomics, it is possible to not only identify specific acetylation sites but also quantitatively characterize the acetylome and determine how relative protein acetylation abundances change dynamically under various treatments and in genetic mutants. Precise and robust quantification of acetylation sites can be achieved by workflows using stable isotope labeling strategies, e.g., metabolic labeling strategies of bacterial strains *in vivo* (SILAC) (152) or chemical labeling strategies of digested protein lysates from different strains using isobaric tags (TMT or iTRAQ) (1, 153). More recently, label-free quantification approaches, such as data-dependent acquisitions in combination with so-called MS1 filtering (49, 154), as well as quantifying PTM sites using data-independent acquisition (76, 155) have become efficient tools for PTM and acetylation site quantification. Other studies have used highly quantitative and targeted approaches applying selected reaction monitoring to monitor acetylation status, such as a recent study assessing specificity and selectivity of Gcn5-mediated acetylation of histone H3 (104).

**(iii) High-resolution mass spectrometric studies provide insight into acetylation site specificity.** Mass spectrometry has been used to extensively profile both enzymatic and nonenzymatic protein acetylation in both eukaryotic and prokaryotic cells. In bacteria, mass spectrometric approaches were applied first and most extensively to E. coli (156, 157). Mass spectrometry was then used to identify enzyme-regulated protein acetylation sites in E. coli mutants lacking YfiQ or CobB (48, 49, 66, 114, 143, 158). Mass spectrometry was also used to investigate nonenzymatic acetylation, comparing mutants that accumulate AcP (*ackA*) to those that lack it (*pta ackA*) (49, 50), comparing WT cells growing in high versus low glucose (130), and comparing WT cells using glucose or xylose as the carbon source (159). Mass spectrometry was recently used to characterize a suite of novel, highly specific KATs and their acetylation profiles (76).

Most mass spectrometry-based workflows take advantage of immunoaffinity purification using anti-K^ac^ antibodies for enrichment of acetylated proteins or, in most cases, the enrichment of acetylated peptides following protein digestion (156, 157). However, some studies analyze acetylation sites directly from bacterial protein lysates without prior PTM enrichment. For example, Nakayasu and coworkers (3) recently used this approach to investigate the proteomes and acetylomes of 48 phylogenetically distant bacteria. Overall, they identified a total of 9,107 acetylated proteins (averaging ∼190 per organism) and 24,397 total acetylated peptides (∼508 per organism). Most importantly, this study revealed acetylation to be a highly conserved and ancient PTM (3). Regardless of workflow strategy with or without PTM enrichment, mass spectrometry is a powerful tool that has been used to identify many acetylation sites on proteins in virtually every cellular process, including central metabolism, translation, and transcription (49, 50, 66, 114, 130, 143, 158, 160–162), and has been used to explore protein acetylation across phylogenetically diverse bacteria (Table 1).

**TABLE 1:** List of bacterial, archaeal, and lower eukaryotic acetylomes

Study (ref. no.)	Organism(s)^*a*^	Mutant(s) assessed	Condition assessed		No. of lysines	No. of proteins
			Time/growth phase^*b*^	Medium		
3	48 bacteria from *Proteobacteria*, *Firmicutes*, *Bacteroidetes*, *Actinobacteria*, *Cyanobacteria*, and *Fibrobacteres*	None	See Table S1 in reference 3	See Table S1 in reference 3	24,397	9,107
196	Acinetobacter baumannii ATCC 17978	None	SP	MHB	551	411
197	Aspergillus flavus CA43 (fungus)	None	48 h	PDA-cellophane	1,383	652
198	Bacillus amyloliquefaciens DSM7	None	EP	LB	3,268	1,254
199	Bacillus nematocida B16	None	12 h	Solid LB with or without nematode incubation	529	349
200	Bacillus subtilis 168	None	SP	LB	332	185
111	Bacillus subtilis 168	None	0.5 OD (EP)	Minimal medium with glucose	1,355	629
201	Bacillus subtilis 168	None	Multiple conditions from previous mass spectrometry runs	Multiple conditions from previous mass spectrometry runs	4,893	1,277
6	Bacillus subtilis 3610	*pta*, *acuA*	SP	LB with 1% (vol/vol) glycerol and 100 μM manganese	1,172	826
164	Bacillus subtilis BD630	None	EP and SP	Minimal glucose medium	2,372	841
186	Borrelia burgdorferi B31-A3	*pta*, *ackA*	EP and SP	BSK-II medium	199	68
165	Clostridium acetobutylicum	None	EP, transition, and SP	Defined medium	458	254
166	Corynebacterium glutamicum ATCC 13869	None	9 h	Glutamate-producing medium +/− Tween 40	1,328	288
202	Cyanobacterium *Synechococcus* sp. PCC 7002	None	EP (under various stresses)	A+ medium	1,653	802
203	Erwinia amylovora Ea1189, Ea273	None	SP	MBMA minimal medium	141	96
158	Escherichia coli BL21	*cobB*	SP	2XYT	2,206	899
130	Escherichia coli BW25113	None	EP, transition, early and late SP; late SP	TB7/glucose; TB7	2,813	780
50	Escherichia coli BW25113, BL21, MG1655	*yfiQ*, *cobB*, *ackA*, *pta*	EP and SP; growth arrested	M9/glucose; nitrogen-limited M9/glucose	8,284	1,000
204	Escherichia coli DH10	None	EP	LB	1,070	349
143	Escherichia coli DH10B	None	EP	M9/glucose/lysine/arginine	2,803	782
156	Escherichia coli DH5α	None	EP	LB	138	91
49	Escherichia coli MG1655	*ackA*, *pta ackA*, *cobB*, *yfiQ*	1 OD (EP-SP transition)	TB7 and TB7/glucose	2,730	806
171	Escherichia coli MG1655 and BW25113	MG1655: *cobB*; BW25113: *ackA*, *pta*	EP and SP; EP	M9/glucose/lysine/arginine	3,669	Not stated
157	Escherichia coli W3110	None	EP and SP	LB	125	85
114	Escherichia coli BW25113	*yfiQ*, *cobB*	EP and SP; EP; steady state	Minimal glucose batch; minimal acetate batch; glucose chemostat	2,502	809
205	Geobacillus kaustophilus 7263	None	SP	Difco nutrient broth	253	114
13	Haloferax mediterranei	None	EP	MG medium	1,017	643
206	Mycobacterium abscessus GZ002	None	EP	Middlebrook 7H9 medium	459	289
167	Mycobacterium smegmatis MC2 155	None	EP, early SP, and middle SP	Middlebrook H79 liquid with 10 mM glucose	146	121
207	Mycobacterium tuberculosis H37Ra	None	EP and SP	Middlebrook 7H9 liquid culture medium	226	137
208	Mycobacterium tuberculosis H37Ra	None	EP; 3 wk	Middlebrook 7H9 aerobically; Middlebrook 7H9 anaerobically	441; 111	286; 83
209	Mycobacterium tuberculosis H37Rv	None	EP	Middlebrook 7H9 medium	1,128	658
191	Mycobacterium tuberculosis H37Rv	None	12 days (EP)	7H9 broth aerobically and anaerobically	1,215	679
210	Mycobacterium tuberculosis L7-35, L7-28, and H37Rv	None	32 days	Middlebrook 7H10 plates	141	109
175	Mycoplasma pneumoniae M129	*pnkB*, *hprK* (kinases); *prpC* (phosphatase); Mpn027, Mpn114 (putative acetyltransferases)	EP	Hayflick medium	719	221
163	Neisseria gonorrhoeae 1291	*ackA*	Overnight	IsoVitaleX-supplemented GC broth	2,686	656
211	Porphyromonas gingivalis W50	None	SP	BHI	130	92
212	Pseudomona aeruginosa PA14	None	24 h	Minimal glucose medium	430	320
213	Pseudomonas aeruginosa PA14	None	SP (24 h)	Minimal medium with citrate, glucose, glutamate, or succinate	1,102	522
71	Rhodopseudomonas palustris CGA009	*ldaA srtN*, *ldaA srtN pat*, *ldaA srtN pat katA*	0.5 OD	Photosynthetic medium with benzoate	32	24
214	Saccharomyces cerevisiae BY4742	*rpd3*	EP	Synthetic complete medium	2,878	1,059
215	Saccharopolyspora erythraea NRRL233338	None	EP	TSBY	664	363
168	Salmonella enterica serovar Typhimurium ATCC 13311	Ciprofloxacin resistant vs WT	EP	LB	1,259	631
216	Salmonella enterica serovar Typhimurium LT2 (G2466)	*pat*, *cobB*	EP	M9/glucose and M9/citrate	235	191
217	Spiroplasma eriocheiris TDA-040725-5T	None	EP	R2 medium	2,567	555
218	Staphylococcus aureus 209P	None	24 h	Cell medium	1,361	412
219	Streptococcus pneumoniae D39	None	EP	THY medium	653	392
73	Streptomyces griseus IFO13350	None	SP; sporulation	Liquid TMPD medium; solid YMPD medium	162	134
220	Streptomyces roseosporus NRRL15998	None	EP (3 days)	F10A medium	1,143	667
221	Sulfurospirillum halorespirans DSM 13726	None	Early and late EP	Defined mineral medium	Not stated	640
222	*Synechocystis* sp. PCC 6803	None	EP	BG11 medium	776	513
113	Thermus thermophilus HB8	None	SP	TT broth	197	128
223	Toxoplasma gondii RH strain	None	64–128 parasites/vacuole	Infected hTERT+HFF cells in DMEM	411	274
224	Toxoplasma gondii RH strain	None	95% host lysis	Infected hTERT+HFF cells in DMEM	571	386
225	Trichophyton rubrum (fungal pathogen)	None	Conidia; mycelia	PDA; Sabouraud liquid medium	386; 5,414	285; 2,335
226	Vibrio cholerae V52	None	EP and SP	LB	3,402	1,240
227	Vibrio parahaemolyticus O3:K6	None	8 h	High-salt LB	1,413	656

a Organism species and strains.

b Exponential growth phase (EP) and stationary phase (SP).

**(iv) Stoichiometry—lysine site occupancy of posttranslational modifications.** Although many studies have measured relative acetylation fold changes (6, 48–50, 114, 130, 163–168), these studies did not provide the stoichiometry or occupancy of acetylation sites (i.e., the fraction occupied [acetylated] versus unoccupied [unacetylated]). Stoichiometry assessments are critical to identify acetylation sites that may have appreciable effects on protein function. Recently, several groups have reported methods and workflows for determining lysine acetylation site occupancies that measure the ratio of endogenously acetylated lysine to unmodified lysine (i.e., stoichiometry or occupancy) (158, 169–174). In stoichiometry experiments, mass spectrometric acquisitions determine the ratio of endogenous “light” acetyl groups to stable isotope-labeled “heavy” acetyl groups, the latter of which are generated after cell harvest by quantitative peracetylation of all unmodified lysines *in vitro* (158, 170). Additional mass spectrometry-based strategies have been described recently (169, 171, 173), indicating the high level of interest in the research community not only in assessing which sites within a given protein are acetylated but also in gaining insight into PTM site occupancy. Such knowledge may help to prioritize PTM-modified sites within a given protein for additional functional assessment and phenotypic investigations.

**(v) Interplay and cross talk of posttranslational modifications.** PTM cross talk is an emerging field that examines how the interaction and interplay between multiple posttranslational modifications affect protein function. Several examples of cross talk between acetylation and other modifications, such as phosphorylation, methylation, ubiquitination, and sumoylation, have now been described (44, 175–178). Given that many modifications other than acetylation occur on lysines, including methylation, ubiquitination, sumoylation, and numerous acylation modifications (179, 180), it is not surprising that there may be interactions between these PTMs, even within the same site of lysine modification. For example, Colak et al. (143) identified 2,803 lysine acetylation sites and 2,580 lysine succinylation sites in E. coli, of which 1,520 sites were both acetylated and succinylated.

Investigating PTM cross talk with mass spectrometry requires the comprehensive identification of multiple PTMs in a single protein sample. This can be challenging as large amounts of protein lysate are required for each PTM enrichment, and the time and cost of sample preparation, data acquisition, and analysis scale with the number of PTMs examined. However, recently Basisty et al. described a “one-pot” affinity enrichment method, a protocol for the simultaneous enrichment of acetylated and succinylated peptides from a single sample, followed by a combined data-independent acquisition analysis and quantification of both modifications, thus greatly reducing the protein, sample preparation, instrument time, and processing time required to examine multiple PTMs (181). The emergence of methods to perform comprehensive multi-PTM profiling such as this has opened the door for large-scale, systems biology studies of PTM cross talk in the future.

### The role of identified acetylated lysines.

With the acetylated lysines identified from mass spectrometry, protein structural data, and/or known structure-function relationships, hypotheses can be developed for how acetylation influences protein function. These exploratory data can be generated *de novo*, or because acetylome studies have been performed in many organisms (Table 1), the literature may already indicate that a protein is acetylated in a provocative location.

Whether acetylation affects protein activity can be investigated in multiple ways. For instance, to generally ask whether acetylation would alter protein function with a known *in vitro* assay, the protein can be purified from cells where acetylation is high (e.g., in an Δ*ackA* mutant of E. coli) versus where it is low (e.g., in WT or a Δ*pta* mutant). The caveat to this approach is that the protein may be acetylated on multiple amino acids, so the exact contribution of each acetylation would need to be assessed through methods described below. Alternatively, a protein purified from any system can be acetylated *in vitro* by AcP and compared to the untreated protein. However, this can generate a heterogeneous population of acetylated proteins or cause the protein to precipitate and should be assayed with care. In addition to the caveat above, AcP can phosphorylate certain proteins (i.e., two-component response regulators), so this contribution must also be considered (182).

To more specifically assess how acetylation of a single lysine regulates protein function, two approaches can be employed. In the first approach, sets of three genetic mimics have been used to assess the contribution of a lysine to a particular function or phenotype (183). By mutating the lysine of interest to arginine, the positive charge and approximate size of a lysine are maintained while being refractory to acetylation. Mutating the lysine to glutamine mimics an acetyllysine by neutralizing the positive charge and maintaining a similar structure. Finally, a lysine-to-alanine mutation removes the side chain to assess the functional importance of the side chain itself. The benefit of this approach is that *in vitro* and *in vivo* studies can be performed with these genetically encoded mutants. A caveat is that, in WT cells, the population of the lysine of interest is heterogeneous with respect to acetylation status; however, these genetic mimics yield a population that is 100% “acetylated” or 100% “deacetylated,” which may not be physiologically relevant. Another caveat is that these mutants are only mimics and may not phenocopy a true acetyllysine (55); therefore, results must be interpreted with care.

The second approach is using site-specific noncanonical amino acid incorporation (184). In this method, three plasmids are introduced into a strain. One plasmid carries the gene of interest. In this gene, the codon encoding the lysine of interest is mutated to an amber codon. A second plasmid contains the suppressor tRNA that will recognize the amber codon, and the third plasmid carries an engineered aminoacyl-tRNA synthetase that will charge an acetyllysine onto the suppressor tRNA. Thus, by supplementing this transformed strain with acetyllysine, the cells will produce a near-homogenous population of acetylated isoforms. These acetylated isoforms then can be purified and used for *in vitro* assays. To confirm that the acetyllysine is incorporated, mass spectrometry or antibodies raised to this specific acetyllysine can be used. While this approach can provide the most direct evidence for how an acetyllysine influences protein function, it is not without flaws. Unfortunately, physiological studies cannot be performed because the system lacks a control where the suppressor tRNA is charged with lysine instead of acetyllysine. Thus, currently, only *in vitro* assays can be performed using this method. Additionally, while one lysine being studied is acetylated, this does not preclude the possibility that there is cross talk between acetylation and/or other posttranslational modifications occurring on other amino acids.

### Evolutionary selection of protein structure by acetylation.

Since the last universal common ancestor (LUCA) was recently hypothesized to be a CO_2_ autotroph that produced AcCoA (185), it is very likely this cell had to evolve with the possibility of spontaneous acetylation to its critical metabolic proteins. Acetylation of a critical lysine could be extremely detrimental to protein function, and the inability to remove these acetylations would result in a dead enzyme. Life must have balanced the possibility of disadvantageous acetylation with benign or beneficial acetylations. To minimize detrimental lysine acetylation under a given condition, the cell could either (i) evolve a lysine deacetylase to remove deleterious modifications or (ii) evolve a protein structure to prevent acetylation. Thus, many of the acetylations we detect today could be harmless or beneficial to protein activity due to selective pressures on the enzyme by acetylation. Based on this hypothesis, many but not all acetylations would be benign. This may be one explanation for why many acetylated lysines are not susceptible to deacetylases as shown in E. coli (48).

### Concluding remarks.

The association between acetylation and metabolism is undeniable. Both enzymatic (via AcCoA) and nonenzymatic (via AcCoA or AcP) mechanisms utilize metabolites as donors of acetyl groups. Furthermore, the removal of acetyl groups via sirtuin-dependent deacetylation requires the oxidized form of the NAD^+^/NADH redox pair, NAD^+^, which plays a key role in central metabolism. Central metabolism is usually one of the most highly acetylated cellular pathways, and acetylation of these metabolic enzymes may serve as a feedback mechanism to direct carbon flux (3).

In a few cases, we know that acetylation of a lysine can affect protein function (1, 2, 20, 137, 186–193). However, there remain thousands of acetyllysines without functional assessment. Dissecting the role of individual lysines provides valuable information, but how the consortium of acetylations work together to affect the entire biological system also must be considered. Let us consider E. coli, where acetylation by AcP is global, resulting in modification of hundreds of proteins on mostly surface-exposed lysines. AcP is generated in response to a carbon-rich environment, which could imply that AcP may be used as a sensor for the nutritional status of the environment. This would not be the only example of how central metabolism provides feedback, as the phosphoenolpyruvate (PEP)/pyruvate ratio can dictate when catabolite repression is active (194, 195). Additionally, many of these acetylated proteins are members of complexes: metabolic, transcriptional, and translational. It is tempting to hypothesize that acetylation could disrupt these complexes, to result in reduced or enhanced activity. Indeed, since AcP is made as a response to carbon overflow, perhaps acetylation could serve as a rheostat to tune down the flux of carbon to help E. coli optimize its growth.

Alternatively, acetylation may serve as a carbon source for cells. Acetylation accumulates during stationary phase when carbon is in excess, which is at the same time and under the same conditions that glycogen, an energy storage compound, accumulates. Similarly to growth on acetate, the two carbon subunits from the acetyl groups could be removed and pass through the glyoxylate shunt to provide biomass or the TCA cycle to provide energy for starved cells.

At this point, the roles of global acetylation and of most individual acetyllysines remain to be elucidated. Clearly, cells have evolved enzymes to specifically acetylate or deacetylate target proteins. Thus, while the acetyl donor may be present, acetylation is dictated by expression or activation of a KAT or KDAC in response to certain stimuli. However, acetyllysines that arise via nonenzymatic acetylation can be regulated only by deacetylation by a KDAC or through alteration of metabolism to disfavor accumulation of the acetyl donors. Because AcCoA is essential and AcP is produced during fermentation, cells may have evolved to either use or cope with the acetylation of their proteins. While this and other hypotheses remain to be tested, knowledge gained in future studies will provide critical insight into the effect of acetylation on cellular physiology and the interconnectedness between metabolism and protein structure and function.
